# Smoking initiation is followed by the early acquisition of epigenetic change in cervical epithelium: a longitudinal study

**DOI:** 10.1038/bjc.2011.113

**Published:** 2011-04-12

**Authors:** Y T Ma, S I Collins, L S Young, P G Murray, C B J Woodman

**Affiliations:** 1 Cancer Research UK Institute for Cancer Studies, School of Cancer Sciences, University of Birmingham, Birmingham, Edgbaston, B15 2TT, UK; 2 Cancer Research UK Clinical Trials Unit, School of Cancer Sciences, University of Birmingham, Birmingham, Edgbaston, B15 2TT, UK

**Keywords:** smoking, epigenetic, methylation, cohort study, cervix

## Abstract

**Background:**

To prove a causal link between an epigenetic change and an environmental or behavioural risk factor for a given disease, it is first necessary to show that the onset of exposure precedes the first detection of that epigenetic change in subjects who are still free of disease.

**Methods:**

Towards this end, a cohort of women aged 15–19 years, recruited soon after they first had sexual intercourse, were used to provide sequential observations on the relationship between cigarette smoking and the detection in cervical cytological samples of methylated forms of CDKN2A (p16) using nested methylation-specific polymerase chain reaction.

**Results:**

Among women who remained cytologically normal and who tested negative for human papillomavirus DNA in cervical smears during follow-up, those who first started to smoke during follow-up had an increased risk of acquiring CDKN2A methylation compared with never-smokers (odds ratio=3.67; 95% confidence interval 1.09–12.33; *P*=0.04).

**Conclusion:**

Smoking initiation is associated with the appearance of methylated forms of CDKN2A.

Exposure to certain behavioural and environmental factors during critical periods of development may result in persistent epigenetic changes, which have phenotypic consequences. For example, in humans, cross-sectional studies suggest that prenatal exposure to famine, *in utero* exposure to airborne polycyclic aromatic hydrocarbons, and childhood abuse are associated with aberrant methylation of the insulin-like growth factor *IGF2*, the long-chain acetyl-CoA synthetase *ACSL3*, and the hippocampal glucocorticoid receptor *NR3C1*, respectively (Heijmans *et al*, 2008; McGowan *et al*, 2009; Perera *et al*, 2009). Aberrant methylation of *ACSL3* and *NR3C1* have in turn been associated with an increased risk of childhood asthma and suicide, respectively (McGowan *et al*, 2009; Perera *et al*, 2009).

Early age at smoking initiation is reported to increase the risk of both lung and cervical cancer, independent of smoking duration or intensity (Wiencke *et al*, 1990; Appleby *et al*, 2006). Such an association is biologically plausible because lung tissues continue to grow and develop into early adulthood, whereas the onset of sexual activity in young women is followed by the extensive remodelling of cervical epithelium, a process, which is accelerated by tobacco smoking (Wiencke and Kelsey, 2002; Hwang *et al*, 2009). However, evidence directly linking early age of smoking initiation to specific genetic, or epigenetic, changes is difficult to adduce. Although cross-sectional surveys, restricted to patients with cancer, have shown that the aberrant methylation of tumour suppressor genes (TSG) is associated with various aspects of smoking exposure, such surveys cannot distinguish those epigenetic changes that are a consequence of the disease process from those that are directly attributable to smoking (Kim *et al*, 2003; Marsit *et al*, 2005). More compelling evidence of smoking-induced epigenetic changes is dependent upon demonstrating, in disease-free individuals, that the onset of smoking precedes the detection of such changes. Such evidence requires a longitudinal study design. The accessibility of the cervix uteri, and the acceptability of the sampling process, presents the opportunity for making repeated observations, thus providing an ideal model for the exploration of the determinants of epigenetic changes. We have investigated the relationship between smoking initiation and the first appearance of methylated forms of the TSG *CDKN2A (p16)*, in cervical cytological samples taken from a cohort of young women who were recruited soon after they first had sexual intercourse.

## Materials and methods

The study design and characteristics of the study population have been described elsewhere (Woodman *et al*, 2001). In brief, 2011 women aged 15–19 years were recruited from a single Brook Advisory Centre (a family planning clinic) in Birmingham, UK, between 1988 and 1992, and asked to re-attend at intervals of 6 months; follow-up ended on 31 August 1997. At recruitment, a standardised interview questionnaire was used to construct a detailed social, sexual, and behavioural risk-factor profile, including smoking. Sexual and smoking histories were updated at each follow-up visit. In addition, at each visit, two cervical cytological samples were taken using the same Ayres spatula; the first was used to prepare a smear for immediate cytological evaluation; and the second was placed into 10 ml of phosphate-buffered saline and stored at −80°C for subsequent virological examination. All women in whom a cervical cytological abnormality was identified were immediately referred to a dedicated research clinic for histological examination, irrespective of the severity of that abnormality. Colposcopic and cytological surveillance were maintained in these women, and treatment was postponed, until there was histological evidence of high-grade cervical intraepithelial neoplasia (CIN2 or CIN3), at which point women left the study. The study was approved by the appropriate Research Ethics Committee, and informed oral consent was obtained from all women.

### Study population

**Figure 1:**
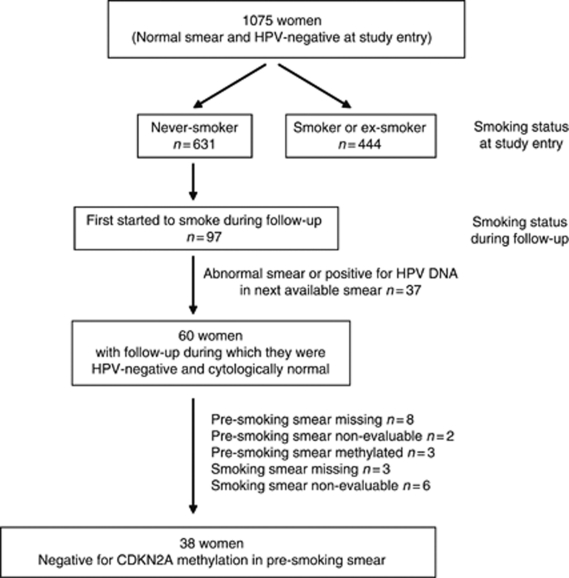
Flow diagram to show the selection of women for *CDKN2A* methylation analysis.

The study population for this analysis was drawn from the 1075 women who were cytologically normal and who tested negative for human papillomavirus (HPV) DNA of any type in cervical samples taken at study entry, and who had further follow-up (Figure 1). From this cohort, we identified 97 women who first began to smoke during follow-up. Of these, 60 women had one or more consecutive samples immediately following smoking initiation when they also tested negative for HPV DNA and were cytologically normal. Excluded from further analyses were: 10 women in whom the last sample taken before smoking initiation was unavailable, or not evaluable; three women in whom this sample tested positive for methylated forms of *CDKN2A*; and nine women in whom the sample taken while they were smoking was unavailable, or not evaluable. The remaining 38 women entered the analysis comparing the risk of *CDKN2A* methylation in incident smokers with that in never-smokers, but only follow-up time before the first detection of HPV DNA of any type, or of cytological abnormality, in cervical samples, was included.

### Assessment of smoking exposure and HPV status

*Assessment of smoking exposure.* Smoking histories were updated at each follow-up visit. Smoking quantity was recorded as a categorical variable, with categories; 0 cigarettes smoked per day, 1–9, 10–19, 20–29, 30–39, and 40 or more. Cumulative smoking exposure was measured using pack-years, estimated as the midpoint of each smoking-quantity category (with the final category arbitrarily set to 45), multiplied by the length of the interval during which this quantity applied, and accumulated over the lifetime of the woman.

*Assessment of HPV status.* Cervical samples were tested for the presence of HPV DNA using a general primer (GP5+/GP6+)-mediated polymerase chain reaction (PCR) and further PCR tests were performed with type-specific primers on samples, which were HPV-positive, using methods previously described (Woodman *et al*, 2001).

### Assessment of outcome status (DNA methylation of *CDKN2A*)

*DNA extraction.* For the present study, the remainder of the stored sample was pelleted, and DNA isolated by digestion with proteinase K, followed by standard phenol–chloroform extraction and ethanol precipitation. The NanoDrop ND-100 Spectrophotometer (Thermo Scientific, Wilmington, DE, USA) was used to measure DNA concentration, according to the manufacturer's instructions.

*Bisulphite modification.* DNA (200 ng) were isolated from each cervical cytological sample, and bisulphite modified using the EZ DNA Methylation-Gold Kit (Zymo Research, Irvine, CA, USA) according to the manufacturer's instructions. The converted DNA was eluted in a final volume of 50 *μ*l.

**Figure 2:**
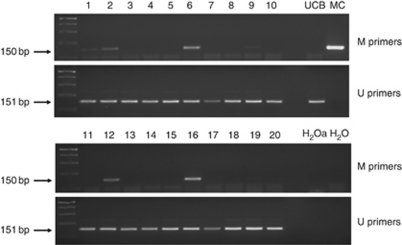
Representative results of the stage 2 PCR using methylated (*M*) and unmethylated (*U*) primers for the *CDKN2A* gene. Lanes 1–20, cervical cytological samples taken from 20 women who were smokers. The presence of a 150 bp product using the methylated primers indicates that the cytological sample was positive for *CDKN2A* methylation (samples 1, 2, 6, 9, 12, and 16). CpGenome Universal Unmethylated DNA (vial B) (UCB) was used as a positive control for unmethylated DNA. CpGenome Universal Methylated DNA (MC) was used as a positive control for methylated DNA. H_2_0a, water from both stages of the nested methylation-specific PCR. H_2_0, water from the stage 2 PCR only.

*Methylation-specific PCR.* As the samples were taken from young women with short smoking histories and were therefore likely to contain only a small number of cells with methylated forms, we elected to test for methylated forms of the *CDKN2A* gene using a nested two-stage methylation-specific PCR method (Palmisano *et al*, 2000). For the stage 1 PCR, 5 *μ*l of bisulphite-modified DNA were amplified in a 25 *μ*l volume using 12.5 *μ*l of GoTaq Green Mastermix (Promega UK Ltd, Southampton, UK), and 0.4 *μ*mol l^−1^ of primer mix. The stage 1 primers recognise the bisulphite-modified template but do not discriminate between methylated and unmethylated alleles. Primer sequences used in this stage were: forward 5′-GAAGAAAGAGGAGGGGTTGG-3′, and reverse 5′-CTACAAACCCTCTACCCACC-3′. Amplification was performed using a P × 2 Thermal Cycler (Thermo Scientific) with the following cycle conditions: 95°C for 10 min, followed by 40 cycles of 95°C for 30 s, 56°C for 30 s, 72°C for 30 s, and a final extension of 72°C for 10 min. The stage 2 PCR was performed on 5 *μ*l of diluted stage 1 PCR product, using primers specific for methylated and unmethylated template. Amplification was performed in a 25 *μ*l volume, using 12.5 *μ*l of GoTaq Green Mastermix (Promega UK Ltd) and 0.4 *μ*mol l^−1^ of primer mix. Primer sequences used in this stage were: forward unmethylated 5′-TTATTAGAGGGTGGGGTGGATTGT-3′, reverse unmethylated 5′-CAACCCCAAACCACAACCATAA-3′, forward methylated 5′-TTATTAGAGGGTGGGGCGGATCGC-3′, and reverse methylated 5′-GACCCCGAACCGCGACCGTAA-3′. For the stage 2 PCR, the annealing temperatures were increased to 65°C (unmethylated) and 70°C (methylated), and all cycling times were reduced to 15 s. All stage 2 amplifications were performed in duplicate. Stage 2 PCR products were analysed using 2% agarose gel electrophoresis and ethidium bromide staining (Figure 2). Controls included CpGenome Universal methylated and unmethylated (vial B) DNA (Millipore (UK) Ltd, Watford, UK). Sensitivity for detecting methylated alleles in a background of unmethylated alleles was determined by mixing DNA isolated from the CpGenome Universal methylated control with DNA isolated from the CpGenome Universal unmethylated control, to achieve dilutions of up to 1 : 100 000. The mixed DNA samples were then subjected to bisulphite modification and subsequent analysis by nested methylation-specific PCR. The sensitivity of the nested methylation-specific PCR assay we used was 1 : 1000, similar to that reported elsewhere (Cirincione *et al*, 2006). To exclude false priming, that is, amplification of unmethylated alleles by the methylated primers, a subset of methylated products were analysed by direct sequencing: methylation of all CpGs within the *CDKN2A* primer sites was observed in every case. A sample was considered to be positive for *CDKN2A* methylation when a methylated band was seen in both stage 2 PCR replicates using the methylated primers; negative for *CDKN2A* methylation when only an unmethylated band was detected; and non-evaluable when no band was seen using either the methylated or the unmethylated primers.

### Statistical analysis

The matched analysis of the incidence of *CDKN2A* methylation was undertaken using conditional logistic regression: confidence intervals for odds ratios were calculated using parameter estimates and their s.e.'s obtained from the model; statistical comparisons were made using the likelihood ratio test. All tests of statistical significance were conducted at the 5% two-sided significance level.

## Results

Of the 38 women who tested negative for *CDKN2A* methylation in the sample taken immediately before they began to smoke, eight tested positive for *CDKN2A* methylation following smoking initiation; the median time to the first detection of *CDKN2A* methylation in those who acquired *CDKN2A* methylation was 266 days (inter-quartile range from 178 to 382). Each of the 38 women contributing to the incidence analysis was matched with two randomly selected never-smokers (controls) on length of follow-up (within 30 days); both groups were similar with respect to demographic and sexual behaviour characteristics (Table 1). For the eight women who first tested positive for methylated forms of *CDKN2A* after smoking initiation, the follow-up period was defined as the time interval between the date of the last unmethylated sample taken before smoking initiation and the date of the first sample to test positive for methylated forms of *CDKN2A* during that smoking episode, that is, while the woman continued to smoke. For the remaining 30 women who did not test positive for methylated forms of *CDKN2A* after they started to smoke, the follow-up period was defined as the time interval between the date of the last unmethylated sample taken before smoking initiation and the date of the last sample taken during that smoking episode. The first and last matched samples from each control were tested for the presence of methylated forms of *CDKN2A*. Three controls were excluded from subsequent analyses because of the presence of methylated forms of *CDKN2A* in the first of their two samples (*n*=2) or because one of the two samples was not evaluable (*n*=1). Of the remaining 73 controls who tested negative for methylated forms of *CDKN2A* in their first sample, five tested positive for these forms in their second sample. Compared with never-smokers, women who first started to smoke during follow-up had an increased risk of the acquisition of methylated forms of *CDKN2A* (odds ratio=3.67; 95% confidence interval 1.09–12.33; *χ*^2^=4.42; 1 degree of freedom; *P*=0.04); controlling for number of sexual partners had no effect.

## Discussion

Using the cervix uteri as a model, we have shown that smoking increases the risk of acquiring methylated forms of *CDKN2A*, a TSG, the epigenetic inactivation of which is strongly associated with the pathogenesis of neoplasia at many sites (Herman *et al*, 1995; Merlo *et al*, 1995). Our longitudinal study design and our unique study population (young women who had recently embarked on sexual activity and who tested negative for HPV DNA of any type and were cytologically free of disease) allowed us to reveal, for the first time, the relationship between an incident exposure (smoking initiation) and the subsequent appearance of an epigenetic change (methylation of *CDKN2A*). Such opportunities for demonstrating temporality *in vivo* come along rarely. The number of women in our original cohort was substantial, but inevitably this was attenuated when analyses were restricted to women who tested negative for HPV DNA of any type and were cytologically free of disease. Nevertheless by recruiting a cohort of women who first began to smoke during follow-up, we were able to show that this epigenetic change appeared soon after smoking initiation. So soon after smoking initiation, in fact, that had our analysis been restricted to women who were prevalent smokers at baseline, we might have failed to detect such an association in a longitudinal analysis in which observations were made at intervals of approximately 6 months.

Our findings are consistent with those of a cross-sectional study reporting a nonsignificant two-fold excess of methylated forms of *CDKN2A* in normal cervical smears taken from smokers, compared with those taken from non-smokers; the higher prevalence of methylated forms of *CDKN2A* observed in our study may be because we used nested methylation-specific PCR, a more sensitive assay (Palmisano *et al*, 2000; Lea *et al*, 2004). Compared with never-smokers, current smokers who are cancer-free, but not necessarily disease-free, are also reported to have a higher prevalence of methylated forms of the TSGs fragile histidine triad (*FHIT*) and *O*-6-methylguanine-DNA methyltransferase (*MGMT*) in bronchial lavages and plasma, respectively (Kim *et al*, 2004; Belinsky *et al*, 2005).

*In vitro* studies in both untransformed and transformed cell lines show that short-term exposure to nicotine, or to cigarette smoke extract, is followed by changes in the expression of the DNA methyltransferases DNMT1, DNMT3A, and DNMT3B. These transcriptional changes have in turn been linked to the hypermethylation of *FHIT*, and to the demethylation of the oncogene synuclein-gamma (*SNCG*) (Soma *et al*, 2006; Liu *et al*, 2007). In murine models of cigarette smoke-induced lung cancer, hypermethylation of the TSGs, death-associated protein kinase 1 (*DAPK1*) and the retinoic acid receptor beta (*RARB*), is seen at the earliest histological stage of adenocarcinoma development (Pulling *et al*, 2004; Vuillemenot *et al*, 2004).

Even allowing for possible inaccuracy in self-reporting, smoking behaviour is unlikely to account for all cases of *CDKN2A* methylation occurring in this cohort. In women who never smoked, epigenetic changes may be explained by other, as yet undefined, risk factors. For example, levels of airborne benzene have been associated with the methylation of *p15* (*CDKN1B*) in healthy individuals (Bollati *et al*, 2007; Baccarelli *et al*, 2009).

Meta-analyses suggest that smoking cessation interventions have little effect on the behaviour of adolescents and young adults (Grimshaw and Stanton, 2006; Myung *et al*, 2009). Adolescent smokers largely discount distant health risks and consider people their own age invulnerable to the adverse effects of smoking (Balch, 1998; McVea *et al*, 2009). It has been argued that new information on the more immediate health effects of smoking is needed to challenge these disengagement beliefs (Kleinjan *et al*, 2009). This study provides such information.

## Figures and Tables

**Table 1:** Baseline characteristics of women in the matched cohort study

**Characteristic**	**Incident smokers (*n*=38)**	**Matched controls (*n*=76)**
Mean age (years)	17.4	17.7
		
*No. of sexual partners (%)*		
0	1 (3)	1 (1)
1	20 (53)	56 (74)
2	12 (32)	7 (9)
3	5 (13)	9 (12)
4	0 (0)	0 (0)
5	0 (0)	1 (1)
>6	0 (0)	2 (3)
Mean no. of sexual partners	1.9	1.1
		
*Age at first sexual intercourse (years) (%)*		
<13	0 (0)	1 (1)
14	4 (11)	3 (4)
15	5 (13)	8 (11)
16	12 (32)	31 (41)
17	10 (26)	19 (25)
18	7 (18)	9 (12)
19	0 (0)	4 (5)
Not applicable	0 (0)	1 (1)
Mean age at first sexual intercourse (years)	16.3	16.5
Mean number of samples tested (range)	2.5 (2–6)	2 (2–2)
